# Altered autonomic nervous system activity as a potential etiological factor of premenstrual syndrome and premenstrual dysphoric disorder

**DOI:** 10.1186/1751-0759-1-24

**Published:** 2007-12-20

**Authors:** Tamaki Matsumoto, Takahisa Ushiroyama, Tetsuya Kimura, Tatsuya Hayashi, Toshio Moritani

**Affiliations:** 1 Department of Health Science, International Buddhist University, 3-2-1 Gakuenmae, Habikino, Osaka, 583-8501, Japan; 2 Department of Nursing, Aino Gakuin College, Ibaragi, Osaka, Japan; 3 Graduate School of Human and Environmental Studies, Kyoto University, Kyoto, Japan

## Abstract

**Background:**

Premenstrual syndrome (PMS) encompasses a wide variety of cyclic and recurrent physical, emotional, and behavioral symptoms occurring during the late luteal phase of the menstrual cycle and abating shortly following the beginning of menses. Although PMS is widely recognized, its etiopathogenesis is not yet understood. The present study investigates whether the activity of the autonomic nervous system, which plays a vital role in orchestrating physiological homeostasis within the human body, is altered during the menstrual cycle of women with different degrees of premenstrual symptomatology.

**Methods:**

Sixty-two women in their 20s to 40s with regular menstrual cycles participated in this study. All subjects were examined during the follicular and late luteal phases. Cycle phase was determined by the onset of menstruation and oral temperature and was verified by concentrations of ovarian hormones, estrone, and pregnanediol in a urine sample taken early in the morning. Autonomic nervous system activity was assessed by means of heart-rate variability (HRV) power spectral analysis during supine rest. The Menstrual Distress Questionnaire was used to evaluate physical, emotional, and behavioral symptoms accompanying the menstrual cycle of the subjects. The subjects were categorized in three groups, Control, PMS, and premenstrual dysphoric disorder (PMDD) groups, depending on the severity of premenstrual symptomatology.

**Results:**

No intramenstrual cycle difference in any of the parameters of HRV was found in the Control group, which had no or a small increase in premenstrual symptoms. In contrast, Total power and high frequency  power, which reflect overall autonomic and parasympathetic nerve activity, respectively, significantly decreased in the late luteal phase from the follicular phase in the PMS group. As for the PMDD group, which had more severe symptoms premenstrually, heart-rate fluctuation as well as all components of the power spectrum of HRV were markedly decreased regardless of the menstrual cycle compared to those of the other two groups.

**Conclusion:**

Several theories have been proposed to explain the underlying mechanisms of PMS with its complex web of bio-psycho-social factors. Although causes and consequences continue to elude, the present study provides intriguing and novel findings that the altered functioning of the autonomic nervous system in the late luteal phase could be associated with diverse psychosomatic and behavioral symptoms appearing premenstrually. In addition, when symptoms become more severe (as seen in women with PMDD), the sympathovagal function might be more depressed regardless of the menstrual cycle.

## Background

Premenstrual syndrome (PMS) is characterized by the cyclic nature of a collection of psychological, physiological, and/or behavioral symptoms appearing during the late luteal phase of the menstrual cycle and usually disappearing shortly after the onset of menses [1,2]. Epidemiological studies, including those in Japan, have shown that up to 90% of women of childbearing age experience at least some degree of premenstrual symptomatology, with an estimated 3–8% having symptoms severe enough to disrupt everyday life and/or interpersonal relationships and to be classified as premenstrual dysphoric disorder (PMDD) [3,4]. Furthermore, according to Kraemer and Kraemer [5], 220 out of 492 women in their study have doctor shopped, seeing an average 3.75 physicians over an average 5.33 years before being diagnosed with PMS and/or effectively treated to alleviate their symptoms.

Notwithstanding this high prevalence, no specific symptoms or signs appear, nor are any recognizable anatomical factors identified in women suffering from PMS, and hence, no universal treatment yet exists. Over the past 70 years, medical and psychological studies have proposed many different theories on the etiology of the premenstrual experience, such as estrogen excess, progesterone deficiency, serotonergic abnormalities, and opioids withdrawal [3,4]. However, the underlying mechanism of PMS, which is multifactorial and might affect diverse neuropsychophysiological systems, remains unclear and speculative.

Sympathetic and parasympathetic divisions of the autonomic nervous system function antagonistically, complementarily, and/or harmoniously to play a crucial role in dynamically controlling the response of the body to a range of external and internal stimuli, maintaining nearly every important homeostatic process in the body. Instability or even a slight disorder of the autonomic nervous system, therefore, could induce broadly ranged psychophysiological phenomena, such as premenstrual symptomatology, and ultimately, far-reaching adverse effects on health. Despite findings that the autonomic function is altered in people with psychosomatic symptoms such as depression [6], anxiety [7], or chronic fatigue [8], a paucity of information exists regarding the potential association of PMS and autonomic nervous system activity.

The electrocardiogram (ECG) sampling of R-R interval variation, or inter-beat interval oscillation of the heart rate is regulated by the net effect of sympathetic and parasympathetic input. The spectral analysis of heart-rate variability (HRV) has provided a comprehensive quantitative and qualitative assessment of sympathetic and parasympathetic components of the autonomic nervous system [9]. In addition, HRV power spectral analysis lightens the burden imposed on subjects during an experiment, unlike invasive measurements, i.e., plasma catecholamine concentration and muscle sympathetic nerve activity. This method, already an established patient-friendly tool in cardiology research, is increasingly used for a broad range of clinical as well as psychophysiological applications [9-13].

Accordingly, the present study evaluates resting autonomic nervous system activity by means of HRV power spectral analysis of eumenorrheic women with different degrees of premenstrual symptoms – from molimina, within the normal range of change, to PMDD, severe PMS [14]. We investigated the extent to which and the manner in which the menstrual cyclicity of autonomic nervous system activity relates to a constellation of diverse symptoms appearing in the late luteal phase.

## Methods

### Subjects

Sixty-two women in their 20s to 40s with regular menstrual cycles participated in this study. The study protocol was approved in advance by the Institutional Review Board of International Buddhist University and was performed in accordance with the Declaration of Helsinki of the World Medical Association. All subjects received an explanation of the nature and purpose of the study, and all gave their written informed consent to enter the study. Prior to obtaining any data from experiments, the subjects had medical examinations and interviews and completed a standardized health questionnaire regarding medical history, medications, current health condition, menstrual cycle (length of cycle, length of menstrual flow, and regularity of cycle), premenstrual discomfort, and lifestyle. All subjects self-reported regular menstrual cycles for at least two cycles. None of the subjects was clinically diagnosed with diabetes mellitus, hypertension, cardiovascular disease, or any other endocrine or systemic disorders.

### Experimental Procedure

All subjects were examined on two separate occasions: once during the follicular phase (within five days after the completion of menstrual bleeding), and once during the late luteal phase (within seven days before the next menstruation). The order of testing during the cycle was randomized to guard against possible bias due to "learning" effects so that equal numbers of subjects were studied first in each phase. Cycle phase was determined by the onset of menstruation and oral temperature and verified by concentrations of ovarian hormones in a urine sample taken early in the morning [13].

All measurements in the follicular and late luteal phase for subjects were taken between 9:00 and 15:00. The room was temperature controlled at 25°C, quiet, and comfortable, with minimization of arousal stimuli. Height and body weight of each subject were measured to calculate body mass index (BMI) as body weight divided by height squared. After skin preparation, the subjects were fitted with ECG electrodes. They then rested for at least 10 minutes before the start of the experiment. After the resting period, the CM5 lead ECG was continuously recorded for five minutes during supine rest. All subjects breathed in synchrony to a metronome at 15 beats·min^-1 ^(0.25 Hz) to ensure that respiratory-linked variations in heart rate did not overlap with lower-frequency heart-rate fluctuations (below 0.15 Hz) from other sources. The ECG sampling of R-R interval variations were later analyzed via HRV power spectral analysis described above to evaluate autonomic nervous system activity during the menstrual cycle.

After the subjects completed ECG measurement, each filled out the Menstrual Distress Questionnaire (MDQ) [15], which evaluated physical, emotional, and behavioral symptoms accompanying the menstrual cycle. The MDQ covers 46 symptoms in eight categories: pain, concentration, behavioral change, autonomic reactions, water retention, negative affect, arousal, and control. The subjects were asked to rate their experience relative to all 46 symptoms on the questionnaire on a six-point scale ranging from no experience of the symptom to so extremely severe as to cause disruption of daily activities.

### R-R interval power spectral analysis procedure

Periodic components of HRV tend to aggregate within several frequency bands [16-18]. The autonomic nervous system activity was thus noninvasively measured by HRV power spectral analysis, which decomposes the series of sequential R-R intervals into a sum of sinusoidal functions of different amplitudes and frequencies by the Fourier transform algorithm. The technique of the analysis for the present investigation has been applied in basic physiological and clinical research fields, and its validity and reliability has been previously confirmed [11-13,19-22]. The procedure of R-R interval power spectral analysis used in the present study has been described in great detail elsewhere [19,20]. Briefly, the ECG signal was amplified (MEG-6108, Nihon Kohden, Tokyo, Japan) and digitized via a 16-bit analog-to-digital converter (Model PS-2032GP, TEAC, Tokyo, Japan) at a sampling rate of 1000 Hz. The digitized ECG signal was differentiated, and the resultant QRS spikes and the intervals of the impulses (R-R intervals) were stored sequentially on a hard disk for later analyses.

Before the R-R spectral analysis was performed, the stored R-R interval data were displayed and aligned sequentially to obtain equally spaced samples with an effective sampling frequency of 2 Hz [23] and displayed on a computer screen for visual inspection. Then, the direct current component and linear trend were completely eliminated by digital filtering for the band-pass between 0.03 and 0.5 Hz. The root mean square value of the R-R interval was calculated as representing the average amplitude. After passing through the Hamming window, power spectral analysis by means of a fast Fourier transform was performed on a consecutive 256-sec time series of R-R interval data obtained during the test. Spectral powers were calculated for the following respective frequency band: low frequency (LF) power (0.03 and 0.15 Hz), an indicator of both sympathetic and parasympathetic nervous system activity; high frequency (HF) power (0.15 and 0.5 Hz), which solely reflects parasympathetic nerve activity; and Total power (0.03 and 0.5 Hz) representing overall ANS activity [11,12,19,20,22].

### Urinary analysis

Each subject collected urine at the first urine void in the morning. Refrigerated 10-mL aliquots of urine were immediately frozen and stored at -20°C until assay. Urine samples were then analyzed for estrone (E1) and pregnanediol-3-glucuronide (PdG) by radioimmunoassay by referring to Munro et al. [24] and De Souza et al. [25]. E1 and PdG were both indexed to creatinine (Cr) excretion in the same sample to control for variations in urine volume. E1 and PdG are expressed as nanograms and micrograms per mg Cr, respectively.

### Statistical analysis

All data are expressed as mean ± SE. Since most of the HRV parameters were not normally distributed as Landen et al. [26] formally suggested, Mann-Whitney U-test was used for comparison between groups, and Wilcoxon Matched-Pairs Signed-Ranks Test for within group comparisons. As to the parameters of symptoms with menstrual cycles and other clinical features, ANOVA using post hoc Tukey's test was used for comparisons between groups, and paired *t*-test for within group comparisons. *P *values < 0.05 were considered statistically significant. All statistical analysis was performed using a commercial software package (SPSS version 13.0 for Windows, SPSS, Inc., Illinois).

## Results

### Clinical characteristics of subjects

Mean values of physical features of all subjects were as follows: age 26.9 ± 1.0 years, height 160.0 ± 0.6 cm, weight 54.8 ± 0.6 kg, and BMI 21.4 ± 0.2 kg/m^2^. Length of menstrual cycle and duration of menstrual flow of subjects during the study were 30.8 ± 0.5 days and 6.3 ± 0.1 days, respectively. The experiments took place on the 8.3 ± 0.3th day in the follicular phase and 28.0 ± 0.6th day in the late luteal phase from the first day of menstruation.

Concerning menstrual cycle symptomatology, some subjects had apparent physical and psychological discomfort in the late luteal phase, while others experienced few symptoms. To scrutinize the potential influence of premenstrual discomfort on autonomic nervous system activity, we first categorized the subjects into two groups based on the increase in scores on the MDQ from the follicular to the late luteal phase while referring to the findings from our recent study [13], i.e., 28 subjects in the Control group (less than 20%) and 23 subjects in the PMS group (greater than 20%). None of the subjects in the PMS group had disruption of normal activities due to premenstrual symptomatology. The remaining 11 subjects, however, experienced distressing psychophysiological and behavioral changes of sufficient severity to result in deterioration of interpersonal relationships and/or interference with daily and/or social activities. They sought medical treatment, according to the results of medical consultation and the MDQ scores. The 11 subjects also met the criteria for PMDD according to the Diagnostic and Statistical Manual of Mental Disorders IV (DSM-IV) [27]. They were, thus, categorized in the PMDD group.

The mean values and range of age in the Control, PMS, and PMDD groups were 25.3 ± 1.3 (20 – 47), 26.7 ± 1.8 (20 – 43), and 31.2 ± 1.9(22 – 43) years, respectively, with no significant difference among the three groups. As to clinical characteristics shown in Table 1, concentration of E1 and PdG in urine was elevated from the follicular to the late luteal phase in all three groups. Group comparison revealed no significant difference in ovarian hormones or in body composition during the menstrual cycle among the three groups.

**Table 1:** Clinical characteristics of subjects in the follicular and late luteal phase

	Control group (n = 28)		PMS group (n = 23)		PMDD group (n = 11)	
	
	Follicular	Luteal	Follicular	Luteal	Follicular	Luteal
Estrone (ng/ml Cr)	10.7 ± 1.0	19.4 ± 2.2**	10.9 ± 1.4	19.3 ± 2.5**	11.3 ± 2.8	21.7 ± 3.5*
Pregnanediol-3-glucuronide (μg/ml Cr)	0.4 ± 0.04	2.1 ± 0.19**	0.3 ± 0.04	2.2 ± 0.3**	0.8 ± 0.4	2.1 ± 0.4
Weight (kg)	54.7 ± 1.2	55.0 ± 1.3	55.9 ± 1.6	56.0 ± 1.6	51.8 ± 2.4	52.6 ± 2.4
Body Mass Index (kg/m^2^)	21.9 ± 0.5	22.0 ± 0.5	21.1 ± 0.5	21.2 ± 0.5	20.5 ± 0.9	20.8 ± 0.9

Cr: creatinine

Values are means ± SE. **p < 0.01; *p < 0.05 (follicular phase vs. late luteal phase)

### Bio-psycho-behavioral symptoms

**Figure 1:**
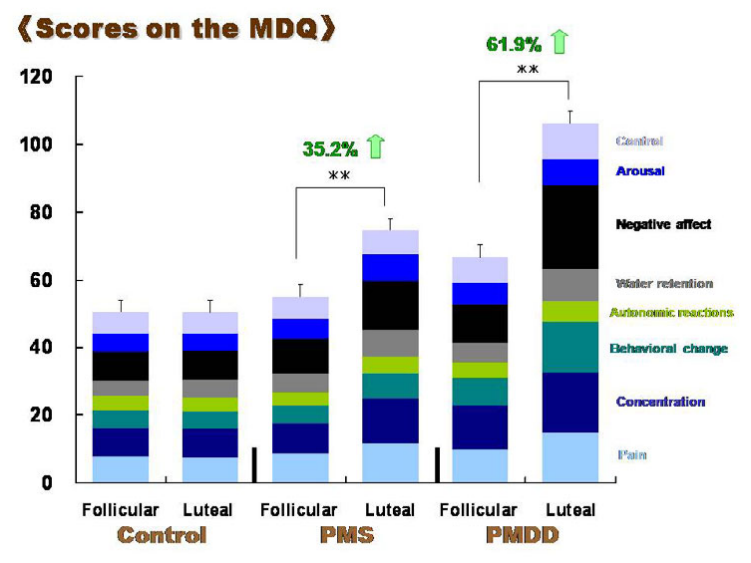
Comparison of scores on the menstrual distress questionnaire (MDQ) between the follicular and late luteal phases in the Control, PMS, and PMDD groups. Results are expressed as mean ± SE. for each group. ** p < 0.01

Figure 1 represents MDQ scores in the follicular and the late luteal phases in all three groups. No significant increase was detected in total scores between the menstrual phases in the Control group. In the PMS and PMDD groups, in contrast, total scores of MDQ in the late luteal phase markedly increased compared to those in the follicular phase (*p *< 0.01). The increase in the MDQ scores from the follicular to the late luteal phase was greater in the PMDD group (61.9 ± 11.8%) than in the PMS group (36.2 ± 3.9%). Scores of all eight factors of MDQ also increased from the follicular to the late luteal phase in the PMS and PMDD groups. The most remarkable change between menstrual phases was detected in negative affect (137.3 ± 36.8%), followed by the scores of behavioral change (93.9 ± 29.1%) and water retention (71.8 ± 13.5%), within the PMDD group. As to the PMS group, subscores of concentration (49.8 ± 9.6%) and negative affect (47.8 ± 8.3%) increased more apparently compared to the other factors.

Group comparison revealed significant differences in total scores on the MDQ in the late luteal phase among the three groups (*p *< 0.01); the scores of the PMS group (74.5 ± 2.4) and PMDD group (106.3 ± 9.5) were approximately 1.5 and 2.1 times greater than that of the Control group (50.3 ± 0.6), respectively. The PMDD group possessed higher scores even in the follicular phase compared to the Control group (66.8 ± 5.9 vs. 50.3 ± 0.7, *p *< 0.01).

### Autonomic nervous system activity

**Figure 2:**
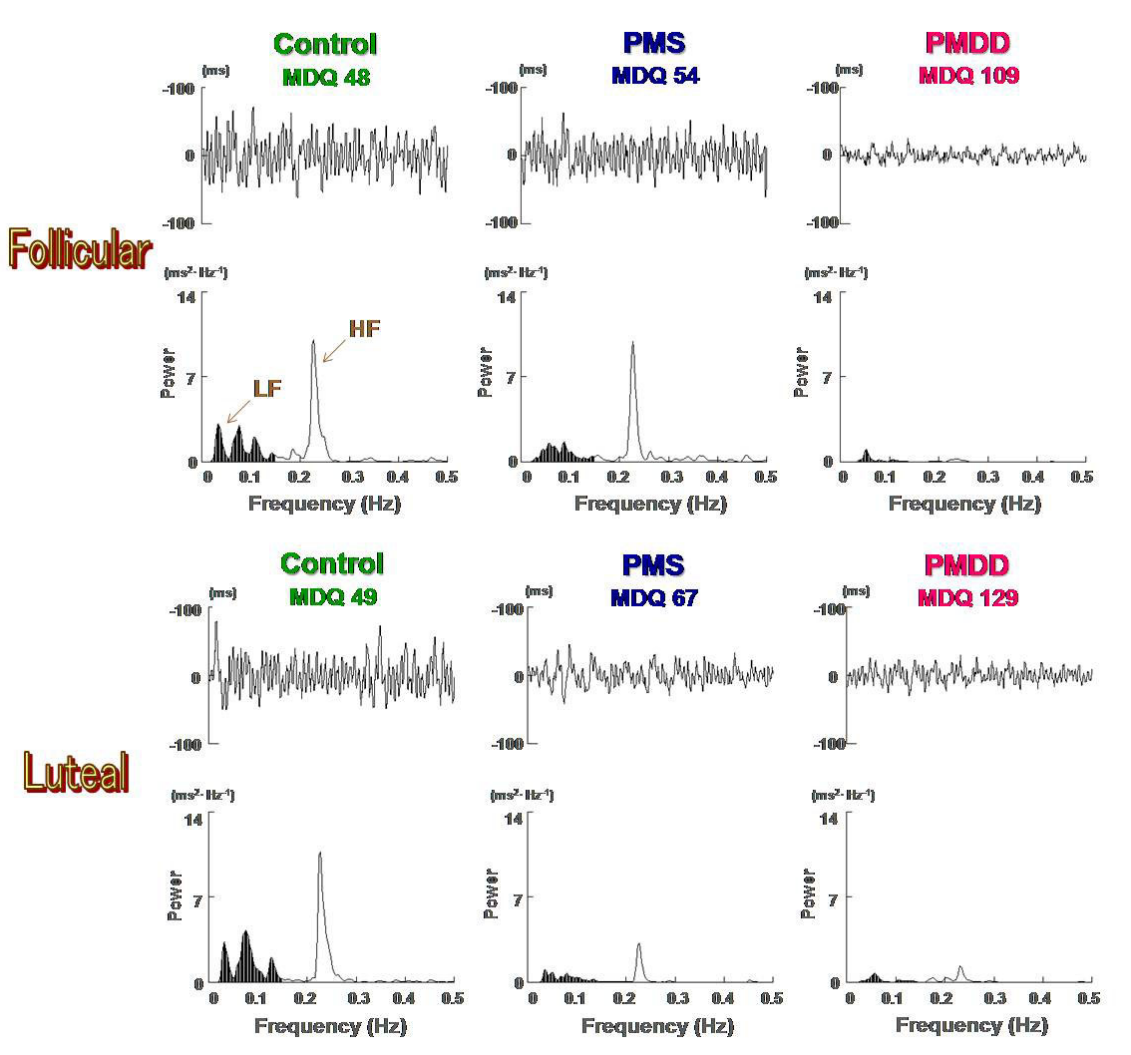
Examples of ECG R-R interval changes and the corresponding power spectra during the follicular and late luteal phase for subjects in the Control, PMS, and PMDD groups. LF: low frequency power (0.03–0.15 Hz); HF: high frequency power (0.15–0.5 Hz).

Figure 2 represents typical sets of raw R-R interval and the corresponding power spectral data obtained from subjects in the Control, PMS, and PMDD groups. We observed widely fluctuated R-R interval and corresponding power spectrum without intramenstrual cycle differences in the Control group subjects. As to the subjects in the PMS group, the HF component of the power spectrum as well as the R-R interval variability decreased in the symptomatic late luteal phase compared to that of the follicular phase. The degree of R-R interval fluctuation and corresponding power spectrum in the follicular phase did not seem to differ between the subjects in the Control and PMS groups. In contrast, the most severe cases we observed among subjects in the PMDD group demonstrate the range of R-R interval variability and spectral power were more markedly reduced regardless of menstrual cycle, compared to the subjects in the Control and PMS groups.

**Figure 3:**
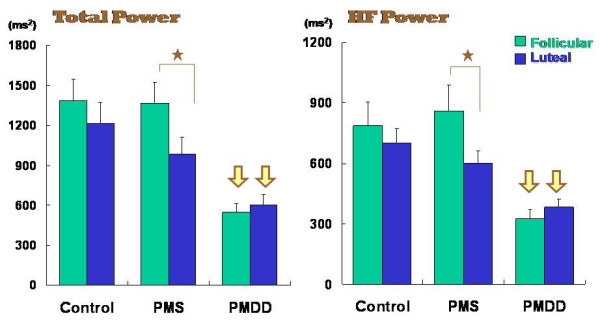
Comparison of Total power and high frequency (HF) power between the follicular and late luteal phases among the Control, PMS, and PMDD groups. Results are expressed as mean ± SE. for each group. p < 0.05 (follicular vs. luteal phases) [star] ; p < 0.05 (Control & PMS group vs. PMDD group) [yellow arrow].

As shown in Figure 3, statistical procedures revealed no differences in any parameters of HRV between the follicular and late luteal phase in the Control group. In the PMS group, however, Total power (*p *< 0.05) and the HF component of the power spectrum (*p *< 0.05) were significantly lower in the symptomatic late luteal phase than in the follicular phase. LF power did not significantly change from the follicular to the late luteal phase in the PMS group. As for the PMDD group, we found obvious reduction in all the power spectral components of HRV in both menstrual phases compared to the other two groups (*p *< 0.05). No intramenstrual cycle difference in any of the components of power was detected in the PMDD group.

## Discussion

PMS comprises myriad nonspecific physical, emotional, behavioral, and cognitive symptoms that occur in the days prior to menstruation and is nearly omnipresent in women of reproductive age from all cultures and socioeconomic levels. The most prevalent symptoms include: irritability, mood lability, depression, anxiety, impulsivity, feelings of "loss of control," fatigue, decreased concentration, abdominal bloating, fluid retention, breast swelling, and general aches [1-4,14]. Although PMS has a multicausal origin and its etiopathogenesis is not yet understood, it is conceivable that altered function of the autonomic nervous system, which largely contributes to the relative stability of a human's internal environment, is associated with the cluster of symptoms appearing premenstrually. The severity and frequency of symptoms experienced may differ between women. The nature of the premenstrual experience is usually stable within each woman but may also fluctuate between her menstrual cycles. This could be attributed to inter-intra individual variability in the change in many aspects of homeostatic functionality during the menstrual cycle [2].

HRV measurements are increasingly used in applications ranging from basic investigations and central regulation of autonomic state to studies of fundamental links between psychological processes and physiological functions and evaluations of cognitive development and clinical risk [9]. According to Kim et al. [28], as an ancillary study of the Women's Health Initiative Observational Study, women with symptoms of depression had significant reductions in HRV and higher heart rate, suggestive of increased sympathetic tone. Yeragani [29], using HRV power spectral analysis, have shown that both Total power and HF power markedly decreased in female patients with depression. Our recent study [22] with this method has revealed that patients with climacteric disorders possessed altered sympathovagal balance. After patient-oriented humanistic psychotherapy, however, unidentified symptoms ameliorated while autonomic imbalance improved with the predominance of parasympathetic nerve activity. These findings demonstrated that power spectral analysis of HRV not only can evaluate the activities of both branches of the autonomic nervous system but also have potential capacity to scrutinize an intricate relationship between psycho-behavioral and physiological processes.

With this bio-fluctuomatics technology, the present study provides intriguing and novel information regarding autonomic nervous system activity during the menstrual cycle in women with different degrees of premenstrual symptomatology. The main findings indicate that no intramenstrual cycle differences in any parameters of HRV were found in the Control group experiencing no or small increases in premenstrual symptoms. It should be mentioned that the widely fluctuated R-R interval and the corresponding power spectrum in the Control group shown in Figure 2 were frequently observed in healthy individuals in our previous research [11,19-21]. In the PMS group, however, Total power and HF power were significantly decreased in the late luteal phase from the follicular phase. As to the PMDD group suffering more severe negative emotional symptoms, heart-rate fluctuation as well as all components of the power spectrum were markedly more reduced regardless of the menstrual cycle compared to the other two groups. Although estrogens and progesterone cannot be excluded as etiological factors of PMS, no significant correlation was detected between urinary ovarian hormone concentration, sympathovagal activity, and premenstrual symptomatology. In addition, age is an important factor influencing autonomic nervous system activity [22,30], but no significant difference was found among the three groups in the present study. Whether the autonomic changes are primary or secondary could not be discerned based on evidence presently available. This study, however, indicates that parasympathetic nervous system activity decreased in the symptomatic late luteal phase compared to the follicular phase in women who experienced a substantial increase (> 20%) in diverse, but not unbearable, psychosomatic symptoms premenstrually. The study also suggests that physiological function in both branches of the autonomic nervous system might be more depressed during the entire menstrual cycle when premenstrual symptoms become more severe as seen in women suffering from PMDD.

We have extensively reviewed the literature regarding the physiological role of the autonomic nervous system in premenstrual symptomatology. Kondo et al. [31] measured the coefficient of variation of R-R interval during the menstrual cycle and demonstrated that the parasympathetic nerve activity was lower in the late luteal phase than in the follicular phase in women with PMS. A recent clinical study by Kimura et al. [32] has applied noninvasive pulse wave analysis to assess the peripheral autonomic reaction as well as arterial elasticity properties and suggested that Kampo therapy could induce vascular rejuvenation, an indication of improvement of autonomic nervous disturbance in PMS patients, while significantly improving premenstrual discomfort. We have utilized the venous oxygenation index (VOI) via the principle of near-infrared spectroscopy to evaluate peripheral blood circulation and demonstrated that the VOI significantly decreased in women with PMS [33]. This result implies altered circulation – at least partly ascribed to autonomic imbalance – is associated with premenstrual symptoms. Although in limited numbers, an association of the autonomic function and PMDD has also been investigated. Girdler et al. [34] revealed that women with PMDD had significantly elevated norepinephrine and total peripheral resistance at rest and during mental stressors compared with control subjects. These phenomena occurred in both the follicular and luteal phases. Recent research by Landen et al. [26] with time and frequency domain of HRV measurement has shown an interesting finding, which suggests that women with PMDD have reduced vagal tone compared to controls and that this difference is more apparent in the non-symptomatic follicular phase.

Despite the differences in experimental designs and conditions as well as an index of the autonomic nervous system, these earlier investigations [26,31-34] support our findings, indicating that the occurrence of premenstrual symptomatology could be attributable to an altered functioning of the autonomic nervous system in the symptomatic late luteal phase. The findings of PMDD from the present study together with Landen et al. [26] and Girdler et al. [34] indicate sympathovagal activity was altered even in the follicular phase. Does this imply that women with lower autonomic function regardless of the menstrual cycle are vulnerable to more severe premenstrual disorders? Clinical research on the menstrual cycle has been conducted from various perspectives, and several theoretical models, such as estrogen excess, progesterone deficiency, decreases in serotonergic tone, alteration of central GABA function, and opioids withdrawal, have been proposed as the pathogenesis of PMS [3,4,14]. However, at the moment, the underlying biomechanisms of PMS remain enigmatic. Future multidirectional and interdisciplinary approaches will be needed to confirm an etiological association of premenstrual symptomatology with the complex web of bio-psycho-social factors around the menstrual cyclicity of sympathovagal function.

## Conclusion

According to Mishell [3], women have approximately 481 menstrual cycles during their childbearing years. With an average adjustment of 22 months for two pregnancies and postpartum periods, they would actually experience about 459 cycles. Regular menstrual cycles could offer an index of women's reproductive health. A majority of women, however, experience "a cacophony of mind and body" in the late luteal phase, that is, premenstrual symptomatology. The present study attempted to investigate these periodical psychophysiological phenomena from the perspective of autonomic nervous system activity with HRV power spectral analysis. Although the underlying pathophysiological mechanisms remain unclear, the present study suggests that altered functioning of the autonomic nervous system in the late luteal phase could be associated with diverse psychosomatic and behavioral symptoms appearing premenstrually. In addition, when the symptoms become more severe, as seen in women with PMDD, the sympathovagal function might be more depressed regardless of the menstrual cycle.

## Competing interests

The author(s) declare that they have no competing interests.

## Authors' contributions

TM^1 ^conceptualized and designed the study, collected and analyzed the data, performed the statistical analysis, interpreted the results, and drafted the manuscript. TU participated in the design and coordination of the present study and provided clinical evidence of PMS and PMDD from his gynecological research as well as practical suggestions to interpret the results. TK contributed to analyzing and diagramming data, helped to interpret the results with productive and valuable comments, and cooperated in promoting the present study. TH participated in the pharmacological experiment to confirm the validity of a method with HRV power spectral analysis, provided endocrinological information for hormonal analysis, and cooperated in developing the present study. TM^3 ^invented a noninvasive monitoring device for HRV power spectral analysis with computer algorithm, and provided constructive and valuable suggestions to interpret the results and to develop the present study. All authors read and approved the final manuscript.
